# Supplementary material for: Endothelial β‐arrestins regulate mechanotransduction by the type II bone morphogenetic protein receptor in primary cilia
Source: Pulm Circ. 2022 Oct 1;12(4):e12167. doi: 10.1002/pul2.12167 (PMC9751664; doi:10.1002/pul2.12167)
Supplement: Supplementary file 1 — Supporting Information. [file PUL2-12-e12167-s001.docx]

**Supplemental Table 1. G protein-coupled receptors and related proteins assessed for localization in primary cilia in endothelial cells.**

| **Receptor** | **Function / Phenotype** | **References** |
| --- | --- | --- |
| **S1PR1**  sphingosine-1-phosphate receptors, EDG1 | - S1P associates with the S1PR1 and regulates angiogenesis and oncogenesis - Genetic disruption of a single S1pr1 gene causes vascular defects | Yu H. et al., 2015, Genes Cells, 20(8):647-58(1)  Karen M. et al., 2013, JBC, 288(4): 2143-2156(2) |
| **CXCR2**  C-X-C motif chemokine receptor2 | - Receptors for angiogenic CXC chemokines - CXCR2 silencing modulates CXCL8-dependent endothelial capillary-like structure formation | Seema S. et al., 2011, Microvasc Res, 82(3): 318-25(3) |
| **CXCR7**  Atypical chemokine receptor 3, ACKR3, C-X-C chemokine receptor type 7 | - Loss of endothelial CXCR7 reduced vascular density - CXCR7 interacts with β-arrestins and are responsible for G-protein-independent signals through ERK1/2 phosphorylation | Circulation, 2017, 135:1253-1264(4)  PLoS One, 2012, 7(3):e34192(5) |
| **CXCR1**  C-X-C motif chemokine receptor1 | - Receptors for angiogenic CXC chemokines - Silencing of CXCR1 inhibited capillary-like structure formation by reducing stress fibers | Seema S. et al., 2011, Microvasc Res, 82(3): 318-25(3) |
| **GPR4**  G-protein-coupled receptor4 | - GPR4 functions as a pH sensor and GPR4-defected mice have vascular abnormalities - Mice lacking GPR4,proton sensing receptor, reduces pathological angiogenesis | Li V. et al., 2007, Mol Cell Biol.(6)  Wyder L. et al., 2011, Angiogenesis(7) |
| **GPR15**  G-protein-coupled receptor15 | - GPR15 mediates angiogenesis and cytoprotective function of thrombomodulin | Pan B., et al., 2017, Sci Rep., 7(1):692(8) |
| **PAR1**  Proteinase-activated receptor1 | - PAR1 is expressed in vascular cells and is involved in atherosclerosis | Nikos E., et al., 2007, Semin Thromb Hemost(9) |
| **RAMP2**  Receptor activity modifying protein2 | - GPCR modulator protein RAMP2 is essential for angiogenesis and vascular integrity - In RAMP2 -/- mice, Vascular abnormalities and reduced responses to angiogenic stimuli | Yuka I., et al., 2008, J Clin Invest 118(10) |


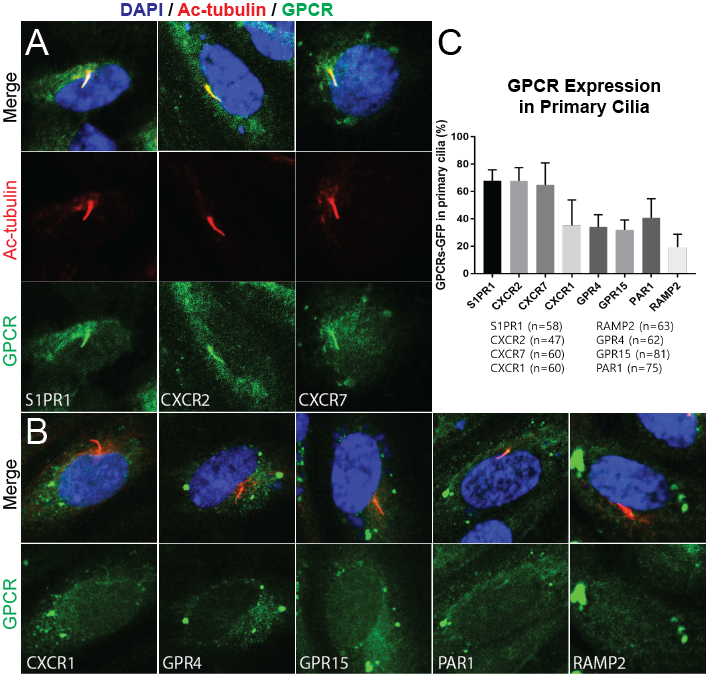


**Supplemental Figure 1. GPCR expression in endothelial cell primary cilia.** (**A**) S1PR1, CXCR2 and CXCR7 (green) are expressed in the primary cilia (Ac-tubulin, red) in the merged image (DAPI, blue). (**B**) No colocalization is observed for other candidate GPCRs and other transmembrane proteins that were tested: CXCR1, GPR4, GPR15, PAR1 and RAMP2. (**C**) Quantification of GPCR GFP signal in primary cilia demonstrates that S1PR1, CXCR2 and CXCR7 are expressed in the primary cilia, while the others do not.


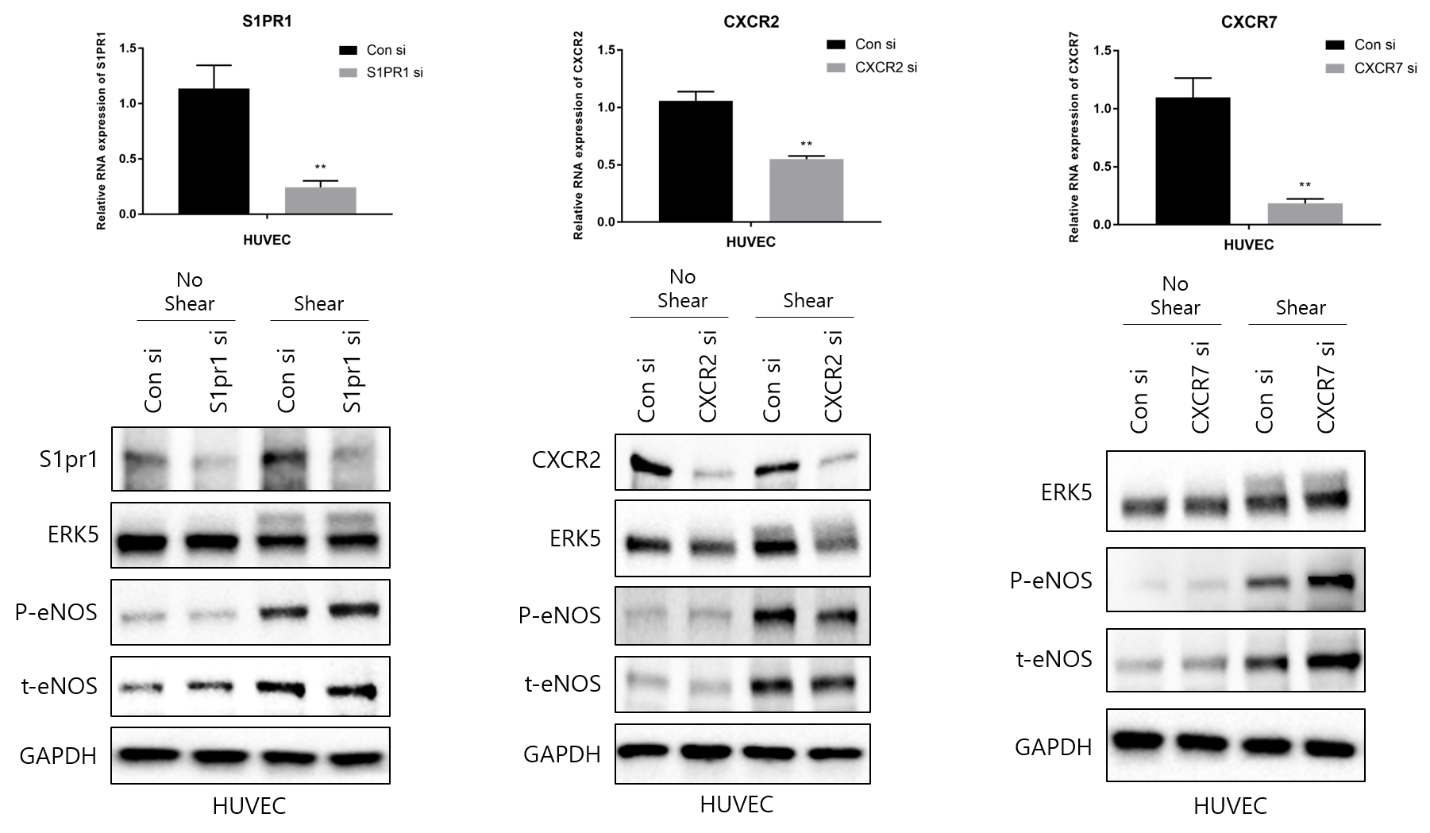


**Supplemental Figure 2. Ciliary GPCR knockdown has no significant effect on eNOS phosphorylation in response to shear.** (**Top panels**) Efficiency of GPCR knockdown. (**Bottom panels**) Knockdown of S1PR1, CXCR3 and CXCR7 had no effect on eNOS phosphorylation in response to shear stress.


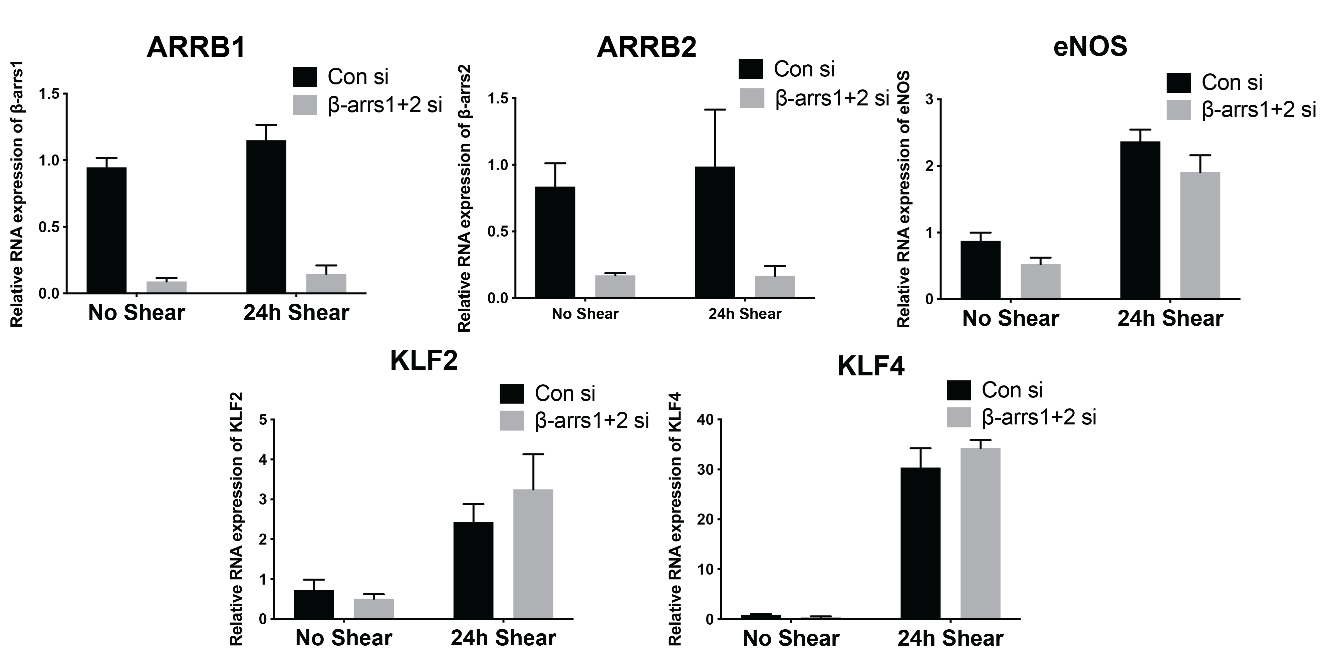


**Supplemental Figure 3. qPCR of targets in response to shear stress in HUVECs.**


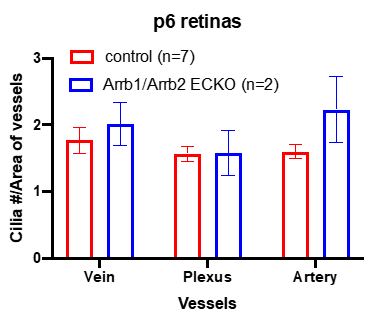


**Supplemental Figure 4.** The number of primary cilia did not differ significantly between vein, plexus, and artery or between control and Arrb1/Arrb2 ECKO retinas.

**References**

1. Hisano Y, Inoue A, Taimatsu K, Ota S, Ohga R, Kotani H, et al. Comprehensive analysis of sphingosine‐1‐phosphate receptor mutants during zebrafish embryogenesis. Genes to Cells. 2015;20(8):647-58.

2. Mendelson K, Zygmunt T, Torres-Vázquez J, Evans T, Hla T. Sphingosine 1-phosphate receptor signaling regulates proper embryonic vascular patterning. Journal of Biological Chemistry. 2013;288(4):2143-56.

3. Singh S, Wu S, Varney M, Singh AP, Singh RK. CXCR1 and CXCR2 silencing modulates CXCL8-dependent endothelial cell proliferation, migration and capillary-like structure formation. Microvascular research. 2011;82(3):318-25.

4. Hao H, Hu S, Chen H, Bu D, Zhu L, Xu C, et al. Loss of endothelial CXCR7 impairs vascular homeostasis and cardiac remodeling after myocardial infarction: implications for cardiovascular drug discovery. Circulation. 2017;135(13):1253-64.

5. Canals M, Scholten DJ, de Munnik S, Han MK, Smit MJ, Leurs R. Ubiquitination of CXCR7 controls receptor trafficking. PloS one. 2012;7(3):e34192.

6. Yang LV, Radu CG, Roy M, Lee S, McLaughlin J, Teitell MA, et al. Vascular abnormalities in mice deficient for the G protein-coupled receptor GPR4 that functions as a pH sensor. Molecular and cellular biology. 2007;27(4):1334-47.

7. Wyder L, Suply T, Ricoux B, Billy E, Schnell C, Baumgarten BU, et al. Reduced pathological angiogenesis and tumor growth in mice lacking GPR4, a proton sensing receptor. Angiogenesis. 2011;14(4):533-44.

8. Pan B, Wang X, Nishioka C, Honda G, Yokoyama A, Zeng L, et al. G-protein coupled receptor 15 mediates angiogenesis and cytoprotective function of thrombomodulin. Scientific reports. 2017;7(1):1-10.

9. Tsopanoglou NE, Maragoudakis ME, editors. Inhibition of angiogenesis by small-molecule antagonists of protease-activated receptor-1. Seminars in thrombosis and hemostasis; 2007: © Thieme Medical Publishers.

10. Ichikawa-Shindo Y, Sakurai T, Kamiyoshi A, Kawate H, Iinuma N, Yoshizawa T, et al. The GPCR modulator protein RAMP2 is essential for angiogenesis and vascular integrity. The Journal of clinical investigation. 2008;118(1):29-39.
